# Combined treatment of disulfiram with PARP inhibitors suppresses ovarian cancer

**DOI:** 10.3389/fonc.2023.1154073

**Published:** 2023-04-18

**Authors:** Bin Tang, Min Wu, Lin Zhang, Shuyi Jian, Shiyi Lv, Tongyuan Lin, Shuangshuang Zhu, Layang Liu, Yixue Wang, Zhengfang Yi, Feiyun Jiang

**Affiliations:** ^1^ Department of Gynecology, East China Normal University Wuhu Affiliated Hospital (The Second People’s Hospital of Wuhu City), Wuhu, China; ^2^ Shanghai Key Laboratory of Regulatory Biology, Institute of Biomedical Sciences and School of Life Sciences, East China Normal University, Shanghai, China

**Keywords:** poly (ADP-ribose) polymerase inhibitors, ovarian cancer, drug combination, disulfiram, DNA damage repair

## Abstract

**Introduction:**

Due to the difficulty of early diagnosis, nearly 70% of ovarian cancer patients are first diagnosed at an advanced stage. Thus, improving current treatment strategies is of great significance for ovarian cancer patients. Fast-developing poly (ADP-ribose) polymerases inhibitors (PARPis) have been beneficial in the treatment of ovarian cancer at different stages of the disease, but PARPis have serious side effects and can result in drug resistance. Using PARPis in combination with other drug therapies could improve the efficacy of PRAPis.In this study, we identified Disulfiram as a potential therapeutic candidate through drug screening and tested its use in combination with PARPis.

**Methods:**

Cytotoxicity tests and colony formation experiments showed that the combination of Disulfiram and PARPis decreased the viability of ovarian cancer cells

**Results:**

The combination of PARPis with Disulfiram also significantly increased the expression of DNA damage index gH2AX and induced more PARP cleavage. In addition, Disulfiram inhibited the expression of genes associated with the DNA damage repair pathway, indicating that Disulfiram functions through the DNA repair pathway.

**Discussion:**

Based on these findings, we propose that Disulfiram reinforces PARPis activity in ovarian cancer cells by improving drug sensitivity. The combined use of Disulfiram and PARPis provides a novel treatment strategy for patients with ovarian cancer.

## Introduction

Ovarian cancer is one of the deadliest gynecological malignancies in the world (1). 90% of ovarian cancers are of an epithelial cell type and comprise multiple histologic types, with various specific molecular changes, clinical behaviours, and treatment outcomes. The remaining 10% are non-epithelial ovarian cancers, which include mainly germ cell tumours, sex cord-stromal tumours, and some extremely rare tumours such as small cell carcinomas (2). In the United States, it is estimated that there were 21,410 new cases and 13,770 deaths in 2021, ranking the fifth highest among female malignancies (3). Approximately 20–30% of epithelial ovarian cancers occur in females with an inherited predisposition; most of these hereditary ovarian cancers are due to germline mutations in BRCA1 and BRCA2 genes. The identification of BRCA1 and BRCA2 pathogenic variants is recommended as an effort of primary prevention for epithelial ovarian cancer (4). Due to the complexity of histological subtypes, biology and clinical features of ovarian cancer, establishing a successful early screening strategy for ovarian cancer is still a major challenge, nearly 70% of ovarian cancer patients are diagnosed at advanced statges, and the 5-year survival rate is only approximately 25%. There are several therapeutic options, such as poly (ADP-ribose) polymerases inhibitors (PARPis), that have been shown to improve ovarian cancer patient survival.

PARPis are the first anti-cancer drugs that successfully applied the synthetic lethal concept. PAPRis have been used to effectively treat Homologous Recombination Deficiency (HRD) tumors (3). Currently, some PARPis, such as Olaparib, Rucaparib, and Niraparib, have been approved for the clinical and maintenance treatment of ovarian cancer (5). They also have been shown to play a very important role in the maintenance treatment of ovarian cancer (6). Based on the 7-year follow-up results of the phase III SOLO1/GOG-3004 trial (NCT01844986) presented at ESMO 2022, Olaparib (Lynparza) maintenance therapy resulted in a long-term overall survival benefit compard to placebo in newly diagnosed advanced ovarian cancer patients with BRCA mutations (7). However, PAPRis treatment is asosociated with serious side effects and drug resistance. To overcome these challenges, PARPis treatment in combination with other targeted drugs, such as Topotecan and Gemcitabine, has been explored (8, 9), but the results are still not satisfactory, and some even produced more serious adverse effects. In a recent phase III trial of Veliparib with platinum therapy, treatment was stopped before determination of disease progression due to high toxicity (10).

Disulfiram has been approved by the FDA and has been widely used in alcoholism treatment for more than 60 years, with low toxicity and controllable side effects (11). Several *in vitro* studies showed that Disulfiram induced apoptosis in cancer cell lines such as breast and ovarian cancer (12). Clinical studies have shown that the main metabolite of Disulfiram, Diethyldithiocarbamate (DDTC), as an adjuvant immunotherapy improves survival of breast cancer patients (13). It was also found that DDTC-copper complex targets NPL4 (the aptamer of separase p97) interferes with the ubiquitinated protease degradation system, inducing cancer cell death. As tumor tissues contain a higher level of copper metabolites than normal tissues, Disulfiram does not cause obvious toxicity for normal cells and has the potential of targeting cancer cells (14).

In this work, we used a drug screen and identified Disulfiram as a potential candidate to be combined with PARPis to treat ovarian cancer. We found that Disulfiram in combination with PARPis synergistically inhibitied ovarian cancer progression, indicating a novel combinatorial treatment strategy for patients with ovarian cancer.

## Materials and methods

### Cell lines, cell culture, and drugs

Ovarian cancer cells SKOV3, ES-2, OVCA420, and HeyA8 were purchased from the American Type Cell Culture (ATCC). SKOV3 cells were cultured in RPMI-1640 medium containing 10% fetal bovine serum (FBS) and 1% penicillin/streptomycin. ES-2, OVCA420, and HeyA8 cells were cultured in DMEM/High Glucose medium containing 10% FBS and 1% penicillin/streptomycin. These cell lines were cultured at 37°C with 5% CO_2_ and 95% humidity. Olaparib (OP) was purchased from MedChemExpress and 100 mM DMSO stock solution was prepared. Disulfiram (DSF) was purchased from Target Molecule, Niraparib (NP) was provided by Zai Lab Co., Ltd., (Shanghai) and a 25 mM stock solution in DMSO was prepared for each drug.

### Cell viability assay and combination index

We seeded SKOV3 and ES-2 cells on 96-well plates at a density of 5×10^3^ cells per well. Cells were treated for 72 h with DMSO, olaparib only (50 μM per well), screening compounds (10 μM per well), and the combination (50 μM Olaparib/10 μM compound per well)., and cell viability was measured using the sulforhodamine B (SRB) assay to determine the relative cell proliferation (15). The SRB test results were analyzed using GraphPad Prism 8 software to calculate the half-inhibition rate of cell proliferation (IC_50_) of the compounds (16), and the results are expressed as the means of triplicate measurements.

According to the IC_50_value of each drug in the cell lines, the final concentration gradient was set as 0.5 IC_50_, 0.75 IC_50_, 1.0 IC_50_, and 1.25 IC_50_. The CI and fraction affected (FA) values were calculated using Calcusyn software, which was based on the Chou-Talalay theorem (17). FA refers to the fraction of cell viability affected. Survivability plots and CI value scatter plots were made in GraphPad Prism 8.

### Colony formation rates

SKOV3 and ES-2 cells were seeded in 6-well cell culture plates in triplicate at a concentration of 5×10^3^ cells per well in 2 mL medium supplemented with 10% FBS and incubated overnight. The media was removed and fresh media containing drugs was added, and the same volume of DMSO was added as a control. The cells were incubated at 37 °C for one week until the colonies were visible to the naked eye. The cells were then fixed with 4% paraformaldehyde for 25 to 30 min, washed with PBS, and stained with 2% crystal violet solution for 15 to 20 min. Finally, the cells were washed with water and air-dried. The number of cell colonies in the wells was counted and the clone formation rate was calculate as: clone formation rate (%)/clone formation rate (control)(%) (18).

### Flow cytometry

SKOV3 cells were seeded in medium containing PARP inhibitors and Disulfiram, and the same volume of DMSO was added as a control. After 48 h of treatment, the supernatants and digested cell suspension were collected. Whole cells in the binding buffer suspension were stained with 1 µL RNA enzyme (Sigma, USA), 2 μL annexin V–FITC (BD, USA), and 2 μL propidium iodide (PI) (Sigma, USA) for 15 min at room temperature in the dark. Unstained cells and single-stained cells were prepared as controls. These samples were detected using flow cytometry, and the stained cells were analyzed using a FACS Calibur (BD). Data were analyzed with FlowJo software (v10).

### Western blot analysis

SKOV3 and ES-2 cells were seeded in 10 cm dishes. Cells were collected after 48 h of drug treatment. Proteins were extracted using RIPA lysis buffer (Sigma) and protein concentrations were determined using the BCA assay. SDS-PAGE (Shanghai Sangon Biological Engineering and Technological Service Company, China) was done according to instruction on the Cell Signaling Technology webstie (19). The following antibodies were used: rabbit anti-PARP antibody (9532s), rabbit anti-γH2AX antibody (9718s), and rabbit anti-GAPDH (ab9485, Abcam). Membranes were scanned using an Odyssey Infrared Imaging System (LI-COR Biosciences), and data were quantified using the Image Studio Lite software.

### Immunofluorescence

Glass coverslips were placed into 24-well plates and 8×10^3^ cells were seeded per well. The cells were treated with Disulfiram and Olaparib at different concentrations and incubated 37°C with 5% CO_2_ and 95% humidity for 48 h. Fixed cells were permeabilized with 0.2% Triton (Sangon, China) in 1×PBS for 30 min. Cells were incubated in 1% BSA (Sangon) in 0.2% Triton/PBS for 30 min. Cells were then incubated with primary rabbit anti-γH2AX antibody (1:400) at 4°C overnight. Cells were then washed with 0.2% triton/PBS three times for 3 min per wash and incubated with a secondary anti-rabbit 800 antibody for 1 h in the dark. Cell nuclei were counterstained with DAPI (D9542, Sigma) for 5 min, and washed with 0.2% triton/PBS three times for 5 min per wash. Images were taken using an Olympus inverted fluorescence microscope.

### Quantitative real-time PCR

SKOV3, ES-2, HeyA8, and OVCA420 cells were treated for 8 h with 15 μM Disulfiram, and total RNA was isolated using the TRIzol reagent (Invitrogen). RNA was extracted and reverse transcribed into cDNA with the Prime Script RT Reagent Kit (Takara). The cDNA was then used as the template for the RT-qPCR reaction that was performed using SYBR-Green (Takara) on QuantStudio^®^3 Real-Time PCR System (Applied Biosystems). GAPDH was used as an internal control. The reaction parameters were as follows: 5 min at 25°C, 30 min at 42°C, 5 min at 85°C, and then held at 16°C. The PCR profile was 95°C for 2 min, followed by 40 cycles of 95°C for 10s and 60°C for 30s. Data were analyzed using GraphPad Prism (version 8; GraphPad Software), and relative gene expression was calculated using the 2-ΔΔCT method.

### Xenograft tumor growth

The ES-2 xenograft tumor models were developed by injecting 1×10^7^ cells into female nude mice (6–8 weeks old). The mice were grouped randomly when the volume of the tumor nodules reached 100 mm^3^ and were then treated with the indicated compounds or vehicle *via* intraperitoneal injection for 18 days. Body weight and tumor dimension were measured. Tumor volume was calculated using the following equation: tumor volume = length × width (2) × 0.52. After the study, the mice were euthanized, and tumors and major organs were collected.

### Immunohistochemistry

Tissue ections were cut from formalin-fixed paraffin-embedded xenografts. For IHC staining, samples were stained using the VECTASTAIN ABC kit (Vector). Anti–Ki67 (1:250; Catalogue #ab15580, Abcam) was used as the primary antibody. Hematoxylin and eosin (HE) staining was performed following standard protocols.

### Statistical analysis

The results are expressed as the mean ± SD. All experiments were performed at least three times, except for the animal experiments. Statistical significance of the difference between two groups was determined by Student’s t-test. Two-way ANOVA was used to analyze animal data. The statistical analyses were performed using GraphPad Prism 7.0. The significant differences in the means were determined at the level of *P < 0.05, **P < 0.01, ***P < 0.001 and ****P < 0.0001.

## Results

### Drug screening in combination with PARPis

**Figure 1:**
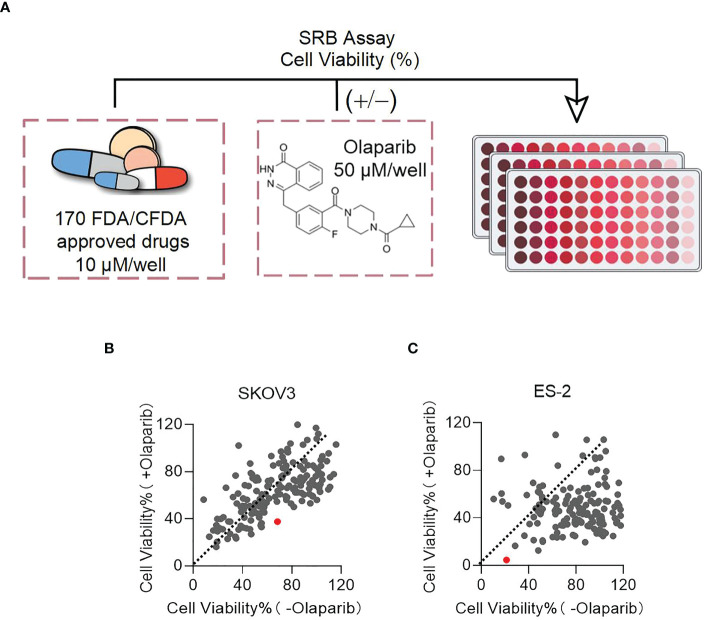
Disulfiram is identified as a target drug to test in combination with PARPis. **(A)** Scheme of the sulforhodamine B (SRB) screening. Plots of cell viability of Disulfiram with Olaparib *vs* without Olaparib in SKOV3 **(B)** and ES-2 cells **(C)**. The dashed line in each figure represents a slope of 1. Each dot represents a compound: red dots represent Disulfiram. Cell viability was determined as described in the Methods section.

To identify drugs that might enhance the effect of PARPis, we screened 170 drug molecules retrieved from the FDA/CFDA compound library using SKOV3 and ES-2 cells. The experiments were performed with the screening compounds alone and the combination with Olaparib. DMSO and Olaparib alone were used as controls. Cell viability of the cells treated with the screening compounds vs the drug combination with Olaparib was plotted (**Figures 1A, B**). In both cases, each dot represents one compound. Dots located below the orange dashed line (slope of 1) indicates that the cell viability ratio of +Olaparib to -Olaparib is below one in the presence of these compounds; the dots located above the dashed line indicated that the cell viability ratio of +Olaparib to -Olaparib is above one. Ninety compounds decreased SKOV3 cell viability, and 151 compounds decreased ES-2 cell viability, indicating that the combinatorial effects of Olaparib were greater in the ES-2 cells (**Figure 1B**). The average survival rate ratios following combined treatment with PARPis *vs* the compound alone were 0.55 (37.61 *vs* 68.22%) and 0.22 (4.58 *vs* 21.28%) for the SKOV3 and ES-2 cells, respectively. Disulfiram (red dot) had the biggest effect on decreasing ES-2 cell viability among the 170 compounds (**Figure 1B**). The chemical structure of Disulfiram is shown in **Figure 1C**.

### Disulfiram in comibation with PARPis inhibited ovarian cell growth

To further confirm the effect of Disulfiram in combination with PARPis, we examined the effect of Disulfiram with PARPis (Olaparib or Niraparib) on cell viability in two additional cell lines, OVCA420 and HEYA8. We first determined the IC_50_ values of Olaparib and Niraparib and in combination with Disulfiram, resepectively. The IC_50_ of Olaparib was higher compared to the IC_50_ of Niraparib, which was relatively low (**Table S1**), consistent with the literature (20). Interestingly, Disulfiram showed relatively low IC_50_ values with in all four cell lines.

**Figure 2:**
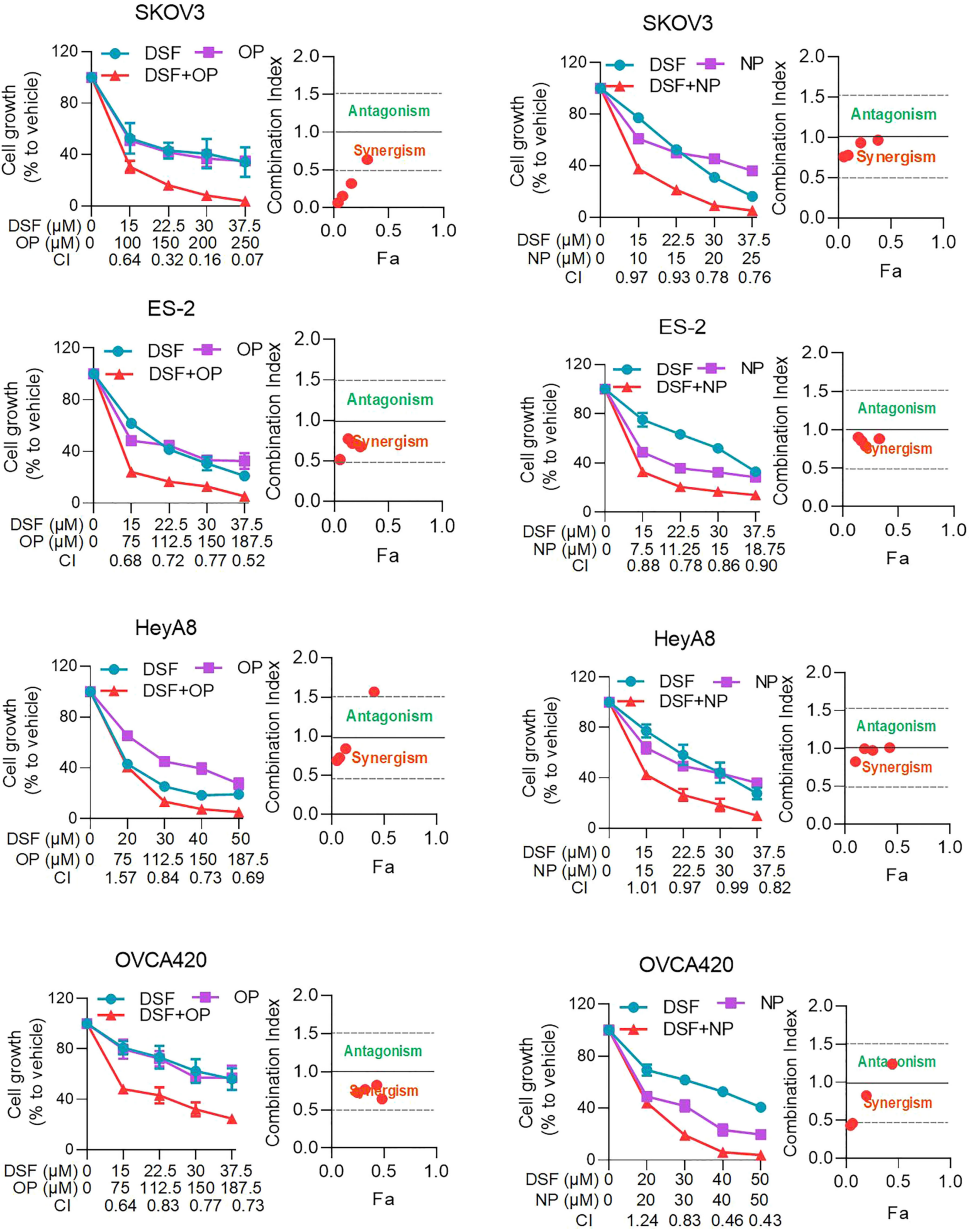
Effect of Disulfiram in combination with and Olaparib and Niraparib on ovarian cancer cell growth. The right column shows the plots of proliferation after treatment with Niraparib and Disulfiram in SKOV3, ES-2, HeyA8, and OVCA420 cell lines. The left column shows the plots of proliferation after treatment with Olaparib and Disulfiram in the SKOV3, ES-2, HeyA8, and OVCA420 cell lines, respectively. The plot of the CI *vs* inhibition rate (Fraction affected, Fa) of each case are shown next to the columns. Dot falls on red “Synergism”indicate a CI below one, and green “Antagonism”indicated a CI above one. Data are represented as mean ± SD (n=4 per group).

We used the CI (combination index) to evaluate whether the effect Disulfiram was additional or synergistic (17). Based on the different IC_50_ values of Olaparib, Niraparib, and Disulfiram (**Figure S1**), we set the concentration of Olaparib or Niraparib with Disulfiram at 50, 75, 100, or 125% of its IC_50_. **Figure 2** shows cell growth at the different concentrations of Olaparib or Niraparib in combination with Disulfram. The green trace of each figure represents the cell growth in the presence of Olaparib (left column) or Niraparib (right column) alone from 0 IC_50_ to 1.25 IC_50_; the purple trace of each figure represents the cell growth in the presence of Disulfiram alone from 0 to 60 µM; and the red trace of each figure represents the cell growth in the presence of Disulfram/Olaparib (left column) and Disulfram/Niraparib (right column) at different concentrations. In all figures, red traces decreased more compared to the corresponding green and purple traces with increasing drug concentrations, indicating that cell growth was more inhibited by the combination of Disulfiram compared to the PARPis alone. The mean CI values of Disulfiram combined with Olaparib or Niraparib in the SKOV3, ES-2, HeyA8, and OVCA420 cells are denoted at the bottom of each figure. The CI values were all below one, indicating that Disulfiram works synergistically with Olaparib or Niraparib to inhibit ovarian cancer cell growth, especially in SKOV3 cells. Moreover, combinational matrix (DSF+OP and DSF+NP) showed effect of Disulfiram in combination with and Olaparib or Niraparib on ovarian cancer cell growth. (**Figures S2A–D**).

**Figure 3:**
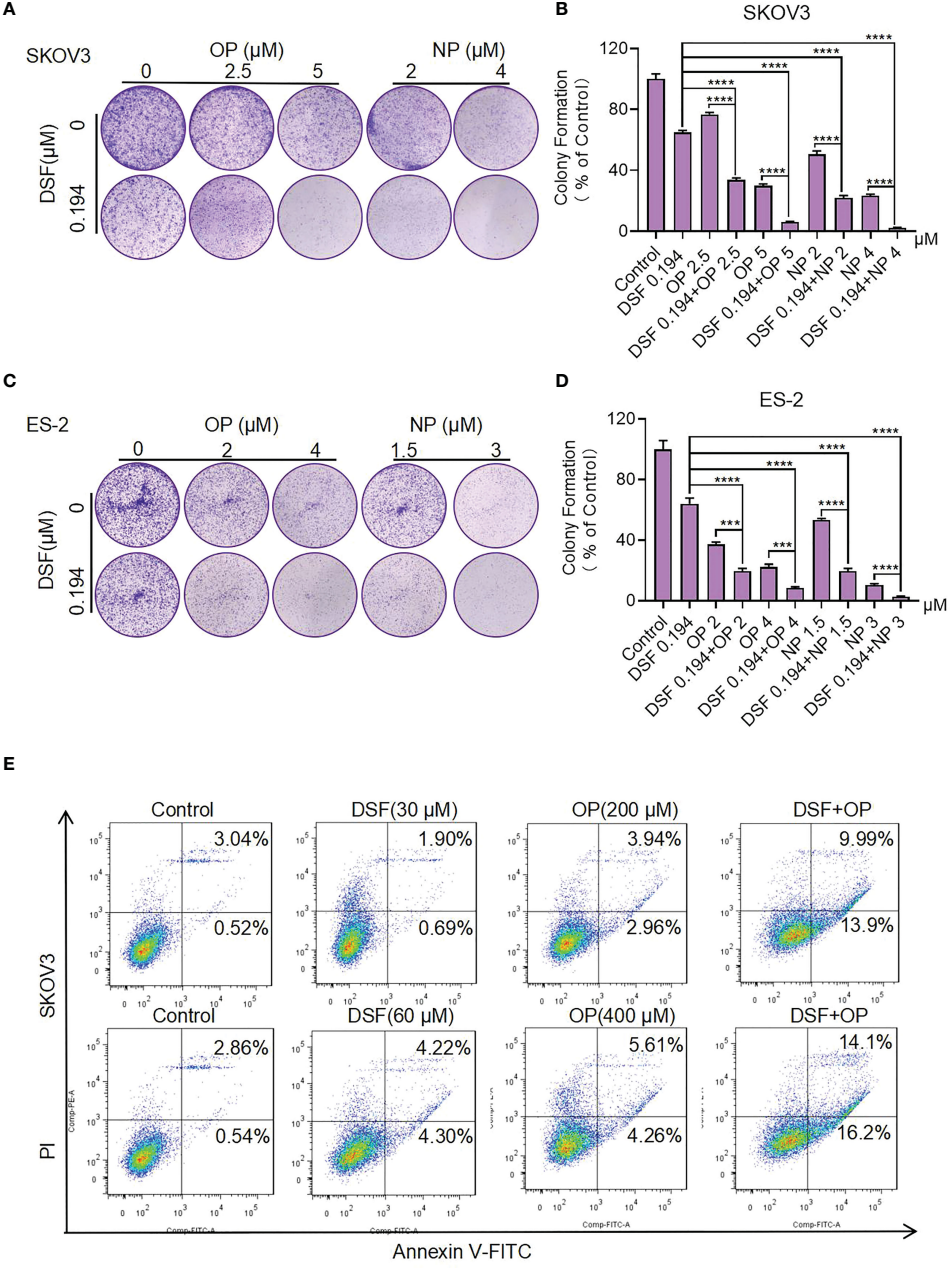
The effect of Disulfiram in combination with PARP inhibitors on colony proliferation and apoptosis of ovarian cancer cells. Clonal Proliferation assay of SKOV3 **(A, B)** and ES-2 cells **(C, D)** treated with the indicated concentrations of Olaparib or Niraparib alone or combined with Disulfiram. Representative images of SKOV3 and ES-2 cell colonies are shown. Apoptosis of SKOV3 cells after treatment with Disulfiram and Olaparib alone and in combination for 48 h at different concentrations **(E)**. Experiments were repeated three times and represented as mean ± SD. Unpaired t-test: ***P<0.001,****P<0.0001.

We further examined the effect of Disulfiram in combination with PARPis on colony formation. Compared with Disulfiram or PARPis alone, the rate of colony formation in the combination group was significantly reduced (**Figure 3**). The average colony formation rate for the combination of Olaparib (5 μM) and Disulfiram (0.194 μM) in SKOV3 cells was reduced to 6.04%, and dropped to 8.33% with Olaparib (4 μM) and Disulfiram (0.194 μM) in ES-2 cells (**Figures 3A–D**). These results are consistent with the findings of cell growth inhibition (**Figure 2**).

### Disulfiram in combination with PARPis increased SKOV3 cell apoptosis

To understand the synergist effect of Disulfiram and PARPis, we measured cell apoptosis of SKOV3 cells after treatment with Disulfiram and Olaparib alone and in combination. After 48 h of drug treatment the total apoptosis rate of Disulfiram (30 µM) with Olaparib (200 µM) was 23.89%, which was approximately 4-fold higher than the apopotosis rate of Olaparib (6.90%) and approximately 10-fold higher than Disulfiram (2.59%) alone. When the concnetration of Disulfiram and Olaparib was doubled, the total apoptosis rate of Disulfiram with Olaparib was 30.3%, approximately 3-fold higher than the apopotosis rate of Olaparib (9.87%) and approximately 3.5-fold higher than Disulfiram (8.52%) alone (**Figure 3E**) and analyzed apopotosis rate (**Figure S3B**). In OVCA420, the proportion of apoptotic cells after drug addition was also detected (**Figure S3A**). Compared with the single drug group, the combination drug induced the generation of apoptotic cells (**Figure S3C**). These results indicate that the combination of Disulfiram with Olaparib increases apoptosis of SKOV3 and OVCA420 cells, which is consistent with the findings for cell growth.

### Disulfiram combined with PARPis increased double-stranded DNA damage.

**Figure 4:**
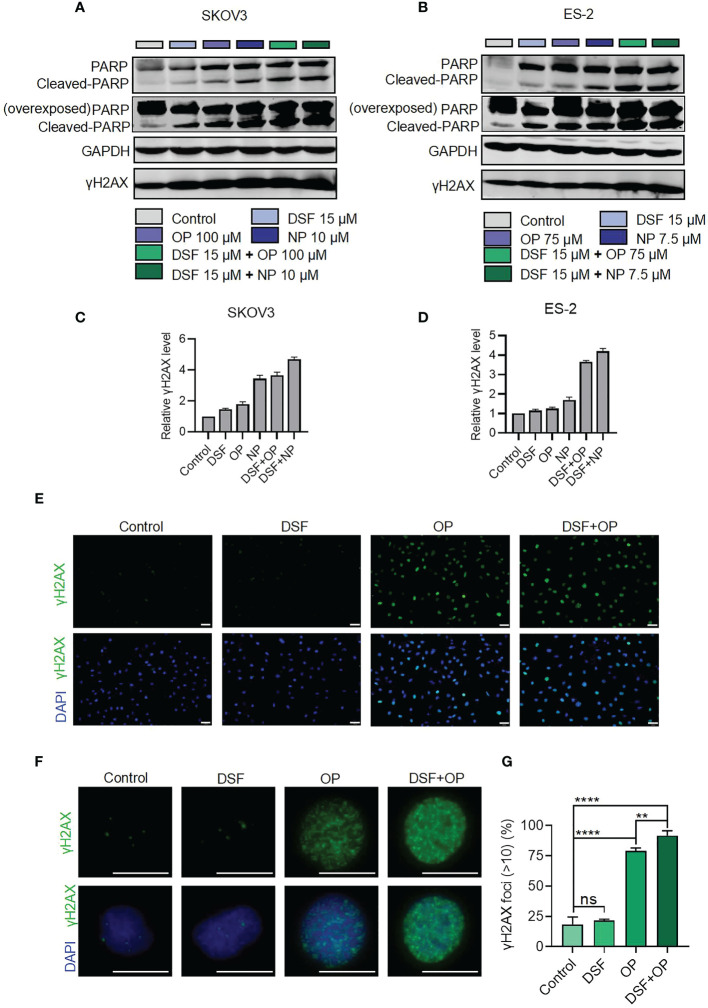
Protein expression of PARP and γH2AX in the presence of Disulfiram and Olaparib and Niraparib. Expression of PARP and γH2AX detected using western blot analysis after treatment with disulfiram and PARP inhibitors alone or in combination in SKOV3 **(A)** and ES-2 cells **(B)**. Densitometry analysis of γH2AX levels normalized to GAPDH in SKOV3 **(C)** and ES-2 **(D)**. Immunofluorescence staining for H2AX in cells treated with Disulfiram (3.75 μM) and Olaparib (25 μM) (E and F). Scale bars are 50 μm **(E)**, and 20 μm **(F)**. Quantification of γH2AX expression **(G)**. The γH2AX foci >10 in all cells in each case were calculated. The experiment was repeated three times. Error bars represent mean ± SD. Unpaired t-test, ns indicates no significant difference, **P<0.01, ****P<0.0001.

As shown in **Figure 3**, the combination of Disulfiram with PARPis increased cell apoptosis. Therefore, we next measured the level of cleaved PARP, which is considered a markers of apoptosis (21), in SKOV3 and ES-2 cells in the presence of Disulfiram and PARPis. We also measured the level of γH2AX (phosphorylation of H2AX, which is one of the most conserved histone H2AX variants), a widely recognized marker of DNA double-strand cleavage (22). **Figures 4A, B** show that Dislfiram in compbination with PARPis increased H2AX protein expression and PARP cleavage compared to PARPis or Disulfiram alone, indicating that the combined treatment increased DNA double-strand cleavage in SKOV3 and ES-2 cells. Apparently, the densitometry analysis showed that the formation of H2AX increased after drug addition or combined drug group in SKOV3 (**Figure 4C**) and ES-2 (**Figure 4D**)

Immunofluorescence assays showed that the percentage of the cells with H2AX foci >10 was 91.72% in the combination group, which was much higher than the Disulfiram (21.67%) or Olaparib (79.05%) alone groups (**Figures 4E–G**). These results confirmed that Disulfiram in combination with Olaparib enhances DNA double-strand damage (**Figure 3**). It is worth noting that the level of DNA damage induced by Disulfiram alone was not significantly different from the Control group (18.46%).

### Disulfiram downregulated genes involved in the homologous recombination repair pathway

**Figure 5:**
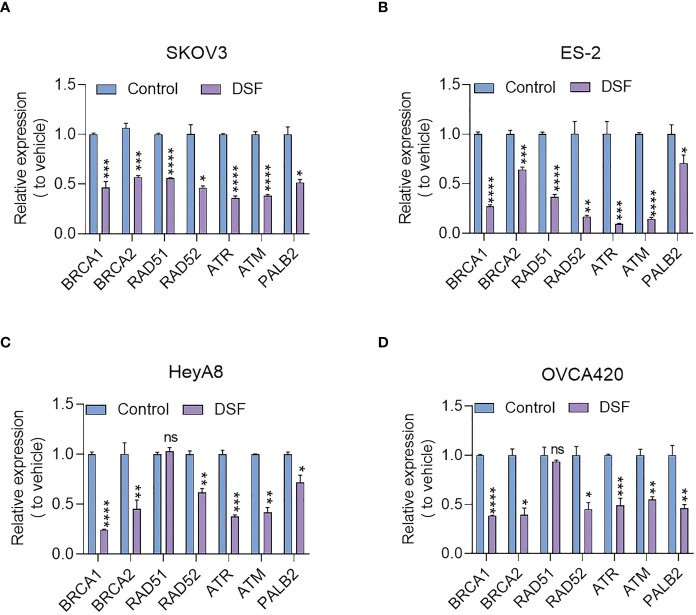
Effect of Disulfiram on expression of HRR-related genes in SKOV3 **(A)**, ES-2 **(B)** HeyA8 **(C)**, and OVCA420 cells **(D)**. The fold-change of inhibition after Disulfiram treatment was plotted in comparison to the control (blue). Each gene is denoted in the Figure. Experiments were repeated three times and represented as mean ± SD. Unpaired t-test, ns, no significant difference; *P<0.05, **P<0.01, ***P<0.001, ****P<0.0001.

HRD cells are more sensitive to PARP inhibitors due to the synthetic lethalilty and because the HRR pathway is not limited to the most common BRCA1/2 mutations. Deletions or mutations in other genes can be directly or indirectly involved in the HRR pathway, which could affect cancer cells sensitivity to PARP inhibitors. We used real-time PCR to determine the transcripton levels of *BRCA1*, *BRCA2*, *RAD51*, *RAD52*, *ATR*, *ATM*, *PALB2*. **Figure 5** shows the expression level of these genes in SKOV3, ES-2, HeyA8, and OVCA420 cells that were treated with 15 μM Disulfiram for 8 h. Compared to the control, Disulfiram significantly inhibited the expression of all-tested genes in the four cell lines, except for *RAD51* in HeyA8 and OZVCA420 cells. These results suggest that the effect of Disulfiram alone on ovarian cancer cells might involve the HRR pathway. However, we did not observe much difference in gene expression in response to the combination of Disulfiram with PARPis *vs* Disulfiram alone (data not shown).

### Disulfiram in combination with Niraparib suppresses growth of ovarian cancer *in vivo*

**Figure 6:**
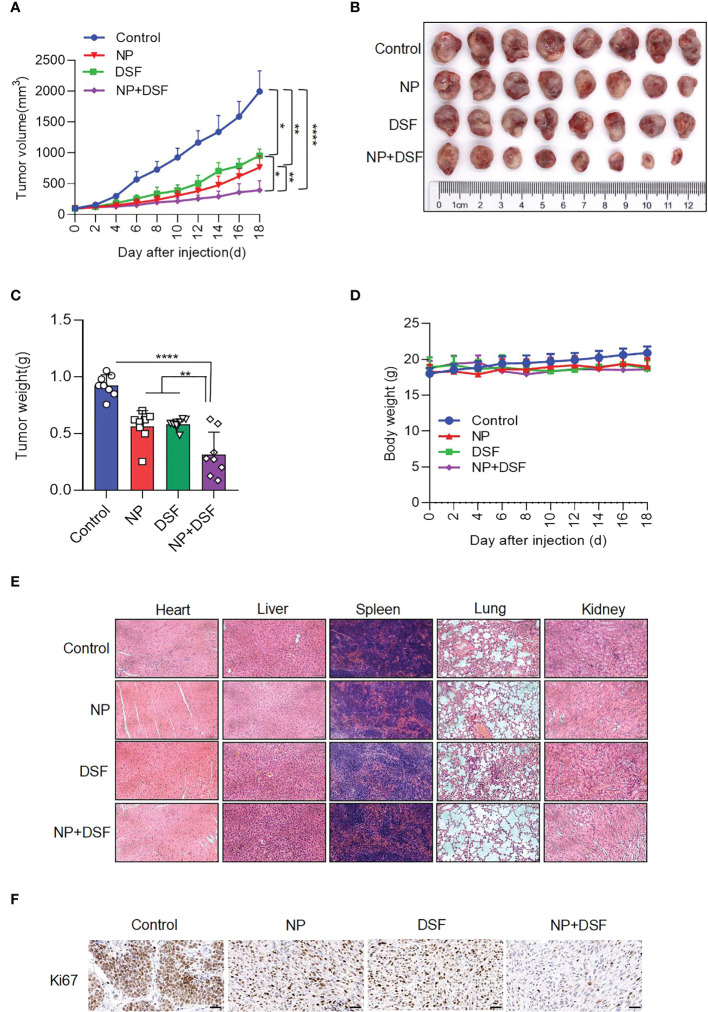
Disulfiram combined with Niraparib suppresses ES-2-derived xenograft tumors *in vivo*. Female nude mice bearing ES-2-derived tumors were randomized into four treatment groups: DMSO, Disulfiram (DSF, 50 mg/kg, daily by i.p.), Niraparib (NP, 50 mg/kg, daily by i.p.), and NP+DSF. After 18 days of treatment **(A)** tumor volumes were measured every 2 days. **(B, C)**. The tumors were photographed and weighed. **(D)** Body weight change was measured **(E)**. HE staining of the heart, liver, lung, kidney, and spleen from the four groups. **(F)** IHC staining of Ki67. Scale bars, 50 µm. *P<0.05; **P<0.01; ****P<0.0001.

Based on the cellular level data, we established an ovarian cancer xenograft model *in vivo* by subcutaneously injecting ES-2 cells into nude mice. **Figure 6** shows that Disulfiram combined with Niraparib suppressed ES-2-derived xenograft tumor growth *in vivo*. Female nude mice bearing ES-2-derived tumors were randomized into four treatment groups: DMSO, Disulfiram, Niraparib, and Niraparib+Disulfiram. **Figures 6A, B** show the change in tumor volume after DMSO (blue trace), Disulfiram (green trace), Niraparib (red trace), and Niraparib+Disulfiram (purple trace) treatment for 18 days. Compared to the DMSO control, both Disulfiram and Niraparib inhibited tumor growth approximately 2-fold after 18 days of the injection, the combination of Disulfiram and Niraparib dramatically inhibited tumor growth compared to the other groups. The change in tumor weight exhibited a similar trend (**Figure 6C**), but the body weight of the mice after the injection of different compounds was very similar across the groups, indicating that Disulfiram, Niraparib, and Niraparib+Disulfiram have negligible toxicity (**Figure 6D**). We did not observe any abnormal behavior or side effects in any of the groups during treatment. This result was confirmed by HE staining of the heart, kidney, lung, liver, and spleen (**Figure 6E**). In immunohistochemistry experiment (23), compared to the control, the proliferation marker Ki67 was dramatically decreased in the Disulfiram+Niraparib group, which confirmed the anticancer effect shown *in vivo* (**Figure 6F**). These *in vivo* data demonstrate that Disulfiram in combination with Niraparib is an effective anti-cancer treatment strategy with minimal to no toxicity.

## Discussion

The proteome closely mirrors the dynamic state of cells, tissues and organisms, proteomics has great potential to deliver clinically relevant biomarkers for ovarian cancer diagnosis. Technologies of proteomics, such as mass spectrometry and protein array analysis, have advanced the dissection of the underlying molecular signaling events and the proteomic characterization of ovarian cancer. Moreover, proteomics analysis of ovarian cancer can uncover new therapeutic choices, which can reduce the emergence of drug resistance (24). Despite rapid developments in cancer diagnosis and precision medicine, ovarian cancer is still recognized as one of most difficult cancers to diagnose early in women, with a high recurrence rate and the highest degree of death (25). PARPis have been successful in prolonging progression-free survival, but tumor recurrence is still inevitable. Some PARPis, such as Olaparib and Niraparib, have already been approved in different settings to treat relapsed epithelial ovarian cancer (26). Combinations of PARP inhibitors with drugs that inhibit homologous recombination may sensitize cancers with a primary or secondary homologous recombination proficiency to PARP inhibitors and potentially expand their use beyond HR-deficient cancers. PARPis in combination with other therapies, such as cytotoxic agents, immunotherapy, and antiangiogenic agents, have shown promising outocmes, and some cases have already been moved to clinical trials. Moreover, PARP inhibitors may be combined separately with PI3K, AKT, mTOR, WEE1, MEK, and CDK4/6 inhibitors (27–29). Studies on the combination of Cediranib (an oral tyrosine kinase inhibitor of vascular endothelial growth factor receptor) with Olaparib have been reported (30). Such combination strategies will open more avenues to optimize the efficacy of PARPis and eventually benefit ovarian cancer patients.We found that the combination of Disulfiram with PARPis has great potential for the development of PARPis combination therapies and expands therapeutic strategies for ovarian cancer.

Disulfiram is an FDA-approved abstinence drug that has advantages of controllable toxicity and side effects and low cost (31). In recent years, many studies have shown that Disulfiram also inhibits cancer progression (32). Here we showed that the anti-cancer activity of Olaparib was greatly enhanced by Disulfiram. We further determined the CI, using the theorm developed by Chou and Talalay, to evaluate the effect of the drug combination. The CI value of each combination case was determined using CompuSyn and Calcusyn software to quantify the synergistic effect of the combined drugs (17). The mean CI values of Disulfiram combined with Olaparib were all below one, which defines synergism. Similar values were found for Disulfiram and Niraparib. Very different CI-*vs*-effect traces were observed for the combinations of Disulfiram/Olaparib and Disulfiram/Niraparib. The combination of Disulfiram/Olaparib showed increasing CI values with increasing effect levels, while the CI values of the combination of Disulfiram/Niraparib were slightly varied with increasing effect levels. This difference in CI values between Niraparib and Olaparib may be attributed to their chemical and physical properties. PARP inhibitors function by trapping PARP1 and PARP2 at DNA lesions, thus abolishing PARylation-mediated DNA damage repair. PARP–DNA complexes have the ability to interfere with DNA replication, and PARP trapping is important for the cytotoxicity of PARP inhibitors. This explains the different magnitude of cytotoxicity exerted by different PARP inhibitors (32, 33).

The combination of Disulfiram and PARPis also increased SKOV3 cell apoptosis. Moreover, a dramatic increase in γH2AX expression level and PARP cleavage in the presence of Disulfiram and PARPis suggest that they work synergistically to cause DNA damage. The combination of Disulfiram with Niraparib suppressed ES-2-derived xenograft tumors *in vivo*, further supporting their synergetic effects. Disulfiram also downregulated the expression of some homologous recombination repair-related genes, such as *BRCA1*, *BRCA2*, *RAD51*, *RAD52*, *ATR*, *ATM*, and *PALB2* in SKOV3, ES-2, OVCA420, and HeyA8 cells (34). Since Disulfiram alone caused no obvious DNA damage (35), these results support that Disulfiram might induce its toxitcity through PARPis in their combnation. In fact, previous work proposed that the anti-cancer activity of Disulfiram was mediated through PARP cleavage, although other mechanisms were proposed as well (36, 37). Because Disulfiram synergistically enhanced the activity of PARPis and affected the genes and proteins associated with DNA damage, we propose that Disulfiram reinforces the activity of PARPis. This reinforcement could be realized by Disulfiram and PARPis individually or through their interaction. It was recently reported that small-molecule p97-complex inhibitors, including a metabolite of Disulfiram, prolonged PARP1 trapping (preventing DNA repair leading to cell death) and enhanced PARP inhibitor-induced cytotoxicity in homologous recombination-defective tumor cells and patient-derived tumor organoids (38, 39). The work proposed that p97 ATPase plays a key role in the processing of trapped PARP1 and the response of tumor cells to PARP inhibitors. Therefore, Disulfiram might increase cell sensitivity to PARPis in a PARP1-dependent manner. Disulfiram likely enhances PARP inhibitor-induced cytotoxicity by inhibiting the normal function of p97 and thereby prolonging PARP1 trapping, which requires further experimentation (39). The variability of the ability of different PARP inhibitors to capture PARP1 (some PARP inhibitors, such as Veliparib, attenuate the interaction between PARP1 and DNA) may explain the difference in the effects of Disulfiram in combination with different PAPR inhibitors (40). The combination of PARPis with other drugs has been suggested to improve anti-cancer efficacy in the clinic for ovarian cancer patients (41). We demonstrated that the combination of Disulfiram and PARP inhibitors expands therapeutic strategies for ovarian cancer patients.

## Data availability statement

The original contributions presented in the study are included in the article/**Supplementary Material**. Further inquiries can be directed to the corresponding authors.

## Ethics statement

All animals were obtained from the Animal Center of East China Normal University. Their care was in accordance with the guidelines of Animal Investigation Committee of the Institute of Biomedical Sciences, East China Normal University.

## Author contributions

BT, MW and LZ contributed equally to this work. BT, LZ, SJ, SL, TL, SZ, LL and YW designed the experiments. BT, LZ, SJ, SL, TL, SZ, LL and YW performed the experiments. BT, LZ, SJ, SL and TL performed the data analysis. BT, LZ, SJ and SL wrote the manuscript. All authors contributed to the article and approved the submitted version.

## References

[1] Vargas AN. Natural history of ovarian cancer. Ecancermedicalscience (2014) 8:465. doi: 10.3332/ecancer.2014.465 25371706 PMC4176445

[2] Cheung A Shah S Parker J Soor P Limbu A Sheriff M. Non-epithelial ovarian cancers: how much do we really know? Int J Environ Res Public Health (2022) 19(3):1-4. doi: 10.3390/ijerph19031106 PMC8834485 35162125

[3] Siegel RL Miller KD Fuchs HE Jemal A. Cancer statistics, 2021. CA: Cancer J Clin (2021) 71(1):7–33. doi: 10.3322/caac.21654 33433946

[4] Shah S Cheung A Kutka M Sheriff M Boussios S. Epithelial ovarian cancer: providing evidence of predisposition genes. Int J Environ Res Public Health (2022) 19(13):1-5. doi: 10.3390/ijerph19138113 PMC9265838 35805770

[5] Curtin NJ Szabo C. Poly(ADP-ribose) polymerase inhibition: past, present and future. Nat Rev Drug Discovery (2020) 19(10):711–36. doi: 10.1038/s41573-020-0076-6 32884152

[6] Cass I Roberts JNT Benoit PR Jensen NV. Multidisciplinary considerations in the maintenance treatment of poly(ADP-ribose) polymerase inhibitors for homologous recombination-proficient, advanced-stage epithelial ovarian cancer. CA: Cancer J Clin (2023) 73(1):8–16. doi: 10.3322/caac.21764 36369877

[7] DiSilvestro P Banerjee S Colombo N Scambia G Kim BG Oaknin A. Overall survival with maintenance olaparib at a 7-year follow-up in patients with newly diagnosed advanced ovarian cancer and a BRCA mutation: the SOLO1/GOG 3004 trial. J Clin Oncol (2023) 41(3):609–17. doi: 10.1200/JCO.22.01549 PMC9870219 36082969

[8] Kummar S Chen A Ji J Zhang Y Reid JM Ames M. Phase I study of PARP inhibitor ABT-888 in combination with topotecan in adults with refractory solid tumors and lymphomas. Cancer Res (2011) 71(17):5626–34. doi: 10.1158/0008-5472.CAN-11-1227 PMC3166628 21795476

[9] Bendell J O'Reilly EM Middleton MR Chau I Hochster H Fielding A. Phase I study of olaparib plus gemcitabine in patients with advanced solid tumours and comparison with gemcitabine alone in patients with locally advanced/metastatic pancreatic cancer. Ann Oncol (2015) 26(4):804–11. doi: 10.1093/annonc/mdu581 PMC8890482 25573533

[10] Diéras V Han HS Kaufman B Wildiers H Friedlander M Ayoub JP. Veliparib with carboplatin and paclitaxel in BRCA-mutated advanced breast cancer (BROCADE3): a randomised, double-blind, placebo-controlled, phase 3 trial. Lancet Oncol (2020) 21(10):1269–82. doi: 10.1016/S1470-2045(20)30447-2 32861273

[11] Johansson B. A review of the pharmacokinetics and pharmacodynamics of disulfiram and its metabolites. Acta Psychiatr Scand Suppl (1992) 369:15–26. doi: 10.1111/j.1600-0447.1992.tb03310.x 1471547

[12] Lu C Li X Ren Y Zhang X. Disulfiram: a novel repurposed drug for cancer therapy. Cancer Chemother Pharmacol (2021) 87(2):159–72. doi: 10.1007/s00280-020-04216-8 33426580

[13] Dufour P Lang JM Giron C Duclos B Haehnel P Jaeck D. Sodium dithiocarb as adjuvant immunotherapy for high risk breast cancer: a randomized study. Biotherapy (1993) 6(1):9–12. doi: 10.1007/BF01877380 8389572

[14] Greco WR Bravo G Parsons JC. The search for synergy: a critical review from a response surface perspective. Pharmacol Rev (1995) 47(2):331–85. 7568331

[15] He XL Xing Y Gu XZ Xiao JX Wang YY Yi Z. The synthesis and antitumor activity of lithocholic acid and its derivatives. Steroids (2017) 125:54–60. doi: 10.1016/j.steroids.2017.06.009 28648585

[16] Chen H Bian A Yang LF Yin X Wang J Ti C. Targeting STAT3 by a small molecule suppresses pancreatic cancer progression. Oncogene (2021) 40(8):1440–57. doi: 10.1038/s41388-020-01626-z PMC7906907 33420372

[17] Chou TC. Theoretical basis, experimental design, and computerized simulation of synergism and antagonism in drug combination studies. Pharmacol Rev (2006) 58(3):621–81. doi: 10.1124/pr.58.3.10 16968952

[18] Cong X He Y Wu H Wang D Liu Y Shao T. Regression of castration-resistant prostate cancer by a novel compound HG122. Front Oncol (2021) 11:650919. doi: 10.3389/fonc.2021.650919 34150618 PMC8210671

[19] Yan B Ai Y Zhang Z Wang X. Assessing POR and CYB5R1 oxidoreductase-mediated oxidative rupture of PUFA in liposomes. STAR Protoc (2021) 2(1):100360. doi: 10.1016/j.xpro.2021.100360 33718888 PMC7933802

[20] Cortez AJ Tudrej P Kujawa KA Lisowska KM. Advances in ovarian cancer therapy. Cancer chemotherapy Pharmacol (2018) 81(1):17–38. doi: 10.1007/s00280-017-3501-8 PMC5754410 29249039

[21] Bonner WM Redon CE Dickey JS Nakamura AJ Sedelnikova OA Solier S. GammaH2AX and cancer. Nat Rev Cancer (2008) 8(12):957–67. doi: 10.1038/nrc2523 PMC3094856 19005492

[22] Kinner A Wu W Staudt C Iliakis G. Gamma-H2AX in recognition and signaling of DNA double-strand breaks in the context of chromatin. Nucleic Acids Res (2008) 36(17):5678–94. doi: 10.1093/nar/gkn550 PMC2553572 18772227

[23] Xing Y Guo W Wu M Xie J Huang D Hu P. An orally available small molecule BCL6 inhibitor effectively suppresses diffuse large b cell lymphoma cells growth *in vitro* and *in vivo* . Cancer Lett (2022) 529:100–11. doi: 10.1016/j.canlet.2021.12.035 34990752

[24] Ghose A Gullapalli SVN Chohan N Bolina A Moschetta M Rassy E. Applications of proteomics in ovarian cancer: dawn of a new era. Proteomes (2022) 10(2):2–6. doi: 10.3390/proteomes10020016 PMC9150001 35645374

[25] Torre LA Trabert B DeSantis CE Miller KD Samimi G Runowicz CD. Ovarian cancer statistics, 2018. CA: Cancer J Clin (2018) 68(4):284–96. doi: 10.3322/caac.21456 PMC6621554 29809280

[26] Konecny GE Kristeleit RS. PARP inhibitors for BRCA1/2-mutated and sporadic ovarian cancer: current practice and future directions. Br J Cancer (2016) 115(10):1157–73. doi: 10.1038/bjc.2016.311 PMC5104889 27736844

[27] Alvarez Secord A O'Malley DM Sood AK Westin SN Liu JF. Rationale for combination PARP inhibitor and antiangiogenic treatment in advanced epithelial ovarian cancer: a review. Gynecologic Oncol (2021) 162(2):482–95. doi: 10.1016/j.ygyno.2021.05.018 34090705

[28] Haynes B Murai J Lee JM. Restored replication fork stabilization, a mechanism of PARP inhibitor resistance, can be overcome by cell cycle checkpoint inhibition. Cancer Treat Rev (2018) 71:1–7. doi: 10.1016/j.ctrv.2018.09.003 30269007 PMC7429716

[29] Revythis A Limbu A Mikropoulos C Ghose A Sanchez E Sheriff M. Recent insights into PARP and immuno-checkpoint inhibitors in epithelial ovarian cancer. Int J Environ Res Public Health (2022) 19(14):1–14 doi: 10.3390/ijerph19148577 PMC9317199 35886427

[30] Mansouri A McGregor N Dunn R Dobbie S Holmes J Collins L. Randomised phase II trial of olaparib, chemotherapy or olaparib and cediranib in patients with platinum-resistant ovarian cancer (OCTOVA): a study protocol. BMJ Open (2021) 11(1):e041463. doi: 10.1136/bmjopen-2020-041463 PMC7813404 33452192

[31] Meier S Cantilena S Niklison Chirou MV Anderson J Hargrave D Salomoni P. Alcohol-abuse drug disulfiram targets pediatric glioma *via* MLL degradation. Cell Death Dis (2021) 12(8):785. doi: 10.1038/s41419-021-04078-9 34381018 PMC8358054

[32] Viola-Rhenals M Patel KR Jaimes-Santamaria L Wu G Liu J Dou QP. Recent advances in antabuse (Disulfiram): the importance of its metal-binding ability to its anticancer activity. Curr medicinal Chem (2018) 25(4):506–24. doi: 10.2174/0929867324666171023161121 PMC6873226 29065820

[33] Boussios S Rassy E Moschetta M Ghose A Adeleke S Sanchez E. BRCA mutations in ovarian and prostate cancer: bench to bedside. Cancers (2022) 14(16):2–16. doi: 10.3390/cancers14163888 PMC9405840 36010882

[34] Marzio A Puccini J Kwon Y Maverakis NK Arbini A Sung P. The f-box domain-dependent activity of EMI1 regulates PARPi sensitivity in triple-negative breast cancers. Mol Cell (2019) 73(2):224–237.e226. doi: 10.1016/j.molcel.2018.11.003 30554948 PMC6995265

[35] Li Z Xie X Tan G Xie F Liu N Li W. Disulfiram synergizes with SRC inhibitors to suppress the growth of pancreatic ductal adenocarcinoma cells *in vitro* and *in vivo* . Biol Pharm Bull (2021) 44(9):1323–31. doi: 10.1248/bpb.b21-00353 34471060

[36] Ekinci E Rohondia S Khan R Dou QP. Repurposing disulfiram as an anti-cancer agent: updated review on literature and patents. Recent patents anti-cancer Drug Discovery (2019) 14(2):113–32. doi: 10.2174/1574892814666190514104035 31084595

[37] Oltersdorf T Elmore SW Shoemaker AR Armstrong RC Augeri DJ Belli BA. An inhibitor of bcl-2 family proteins induces regression of solid tumours. Nature (2005) 435(7042):677–81. doi: 10.1038/nature03579 15902208

[38] Götz MJ Stingele J. Releasing the trap: how the segregase p97 extracts PARP1 from chromatin. Mol Cell (2022) 82(5):889–90. doi: 10.1016/j.molcel.2022.02.012 35245455

[39] Krastev DB Li S Sun Y Wicks AJ Hoslett G Weekes D. The ubiquitin-dependent ATPase p97 removes cytotoxic trapped PARP1 from chromatin. Nat Cell Biol (2022) 24(1):62–73. doi: 10.1038/s41556-021-00807-6 35013556 PMC8760077

[40] Zandarashvili L Langelier MF Velagapudi UK Hancock MA Steffen JD Billur R. Structural basis for allosteric PARP-1 retention on DNA breaks. Sci (New York NY) (2020) 368(6486):2–16 doi: 10.1126/science.aax6367 PMC7347020 32241924

[41] Mittica G Ghisoni E Giannone G Genta S Aglietta M Sapino A. PARP inhibitors in ovarian cancer. Recent patents anti-cancer Drug Discovery (2018) 13(4):392–410. doi: 10.2174/1574892813666180305165256 29512470

