# Supplementary material for: Combined treatment of disulfiram with PARP inhibitors suppresses ovarian cancer
Source: Front Oncol. 2023 Apr 18;13:1154073. doi: 10.3389/fonc.2023.1154073 (PMC10151711; doi:10.3389/fonc.2023.1154073)
Supplement: Supplementary file 2 [file DataSheet_1.docx]

Supplemental Materials for

**A combination of Disulfiram with PARP inhibitors suppresses ovarian cancer**

Bin Tang^1^,^#^ Min Wu^2^,^#^ Lin Zhang^2^,^#^ Shuyi Jian^2^, Shiyi Lv^2^,Tongyuan Lin^1^, Shuangshuang Zhu^2^, Layang Liu^2^,Yixue Wang^2^, Zhengfang Yi^2^* and Feiyun Jiang^1^*

*^1^Department of Gynecology, East China Normal University Wuhu Affiliated Hospital (The Second People's Hospital of Wuhu City), Wuhu 241000, China.*

*^2^Shanghai Key Laboratory of Regulatory Biology, Institute of Biomedical Sciences and School of Life Sciences, East China Normal University, 500 Dong Chuan Rd, Shanghai 200241, China.*

**Corresponding Authors**

* Feiyun Jiang: E-mail: fyjiang6872@163.com

* Zhengfang Yi: E-mail: zfyi@bio.ecnu.edu.cn

**Figure S1**. **Inhibitory activity of Disulfiram and PARPi on proliferation of ovarian cancer cells.** (A-C) Proliferation inhibition curves of Disulfiram, Olaparib and Niraparib in ovarian cancer cell lines SKOV3, ES-2, OVCA420 and HeyA8, the data was expressed as mean±SD. (D) Summary of IC50 values in cell lines.

**
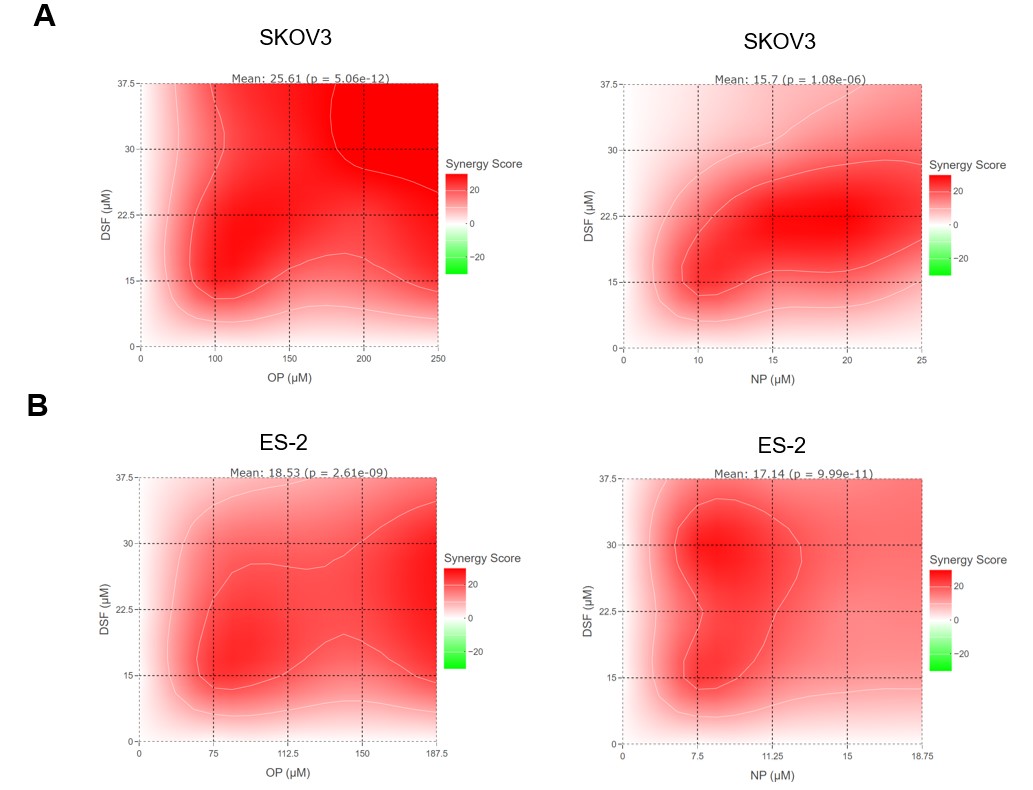
**

**
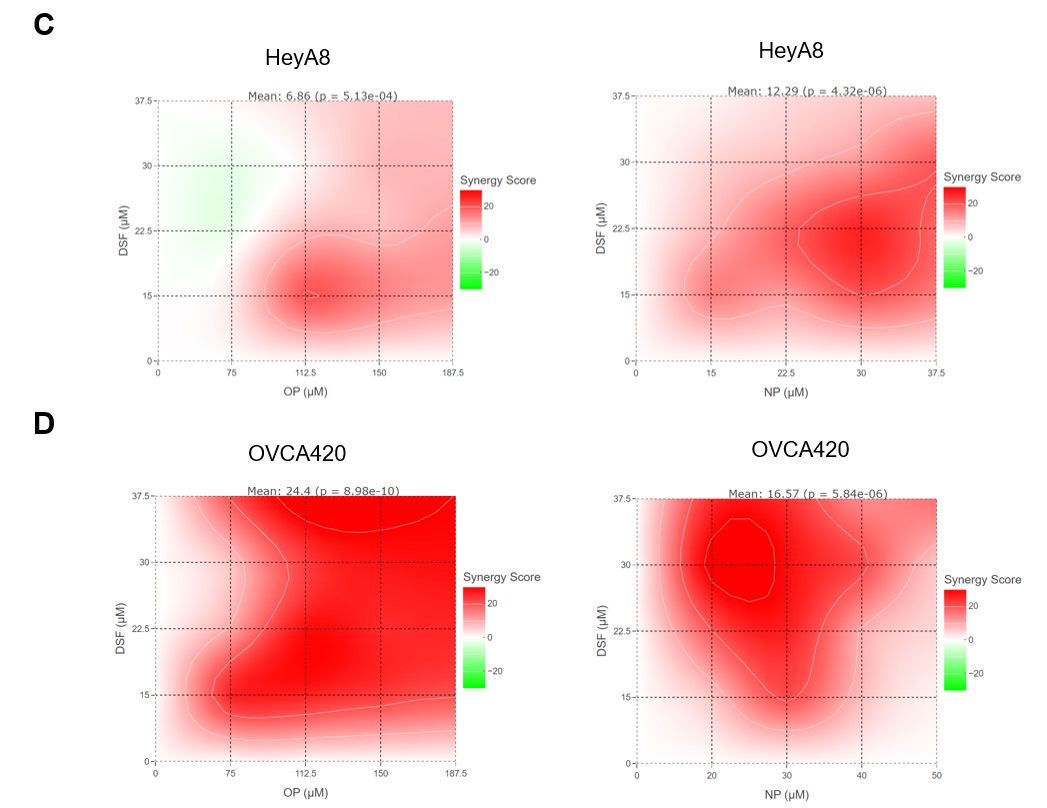
**

**Figure S2.** **Effect of Disulfiram in combination with and Olaparib and Niraparib on ovarian cancer cell growth.** Combinational matrix shows proliferation after treatment with Olaparib or Niraparib and Disulfiram in the SKOV3(A), ES-2(B), HeyA8(C), and OVCA420(D) cell lines. Synergy scores were calculated using Synergyfinder software. HSA (highest single agent) algorithm was used to calculate synergy scores, and color keys indicate the scores in heatmaps.


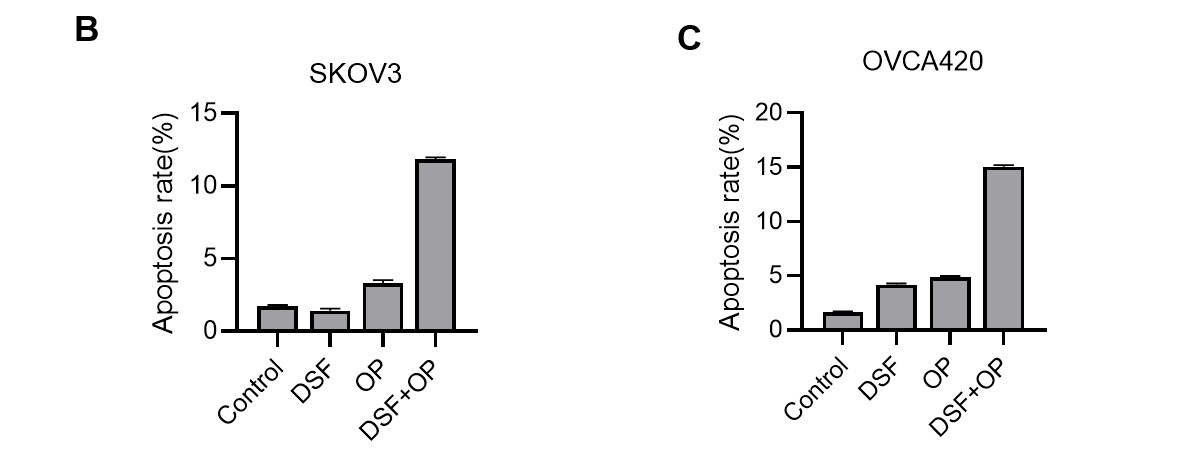

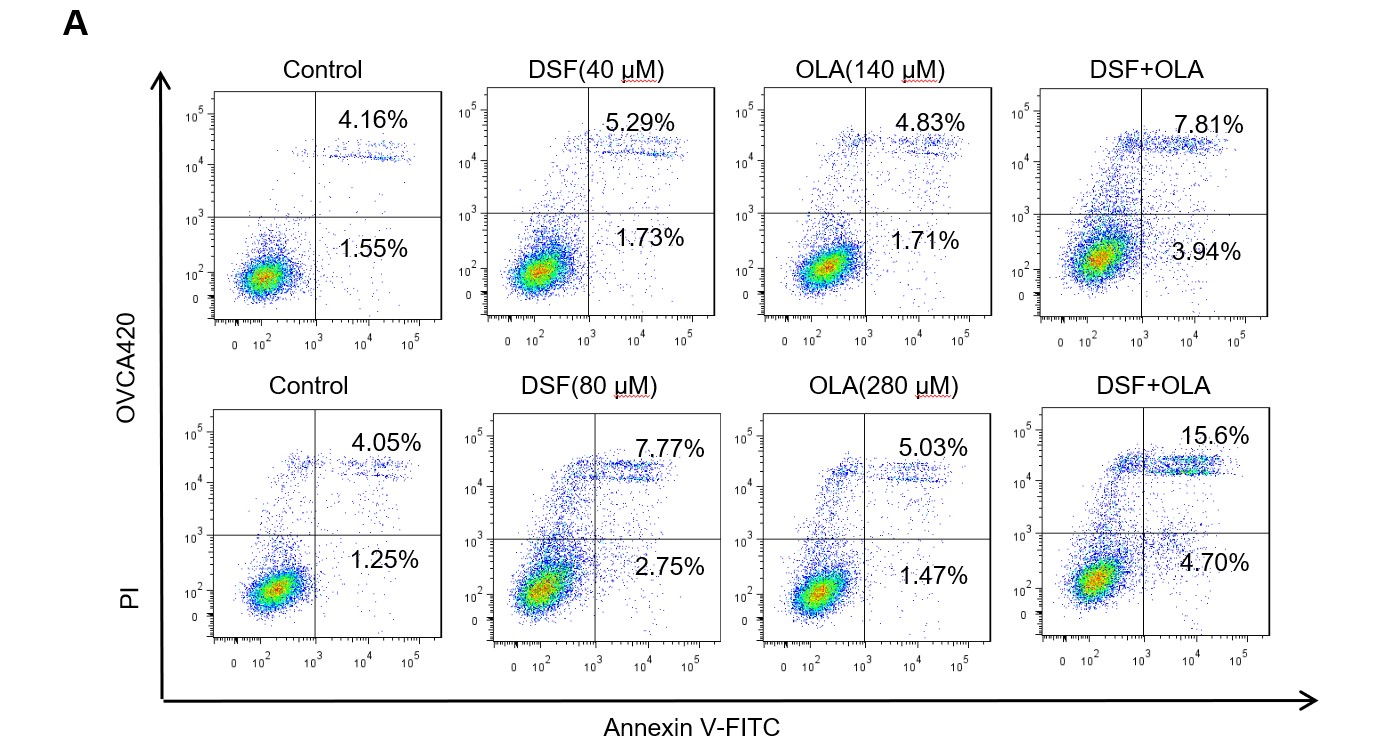


**Figure S3.** **Drug administration induce apoptosis of ovarian cancer cells.** Apoptosis of OVCA420 cells after treatment with Disulfiram and Olaparib alone and in combination for 48 h at different concentrations (A). Statistical analysis of the proportion of apoptotic cells in SKOV3 (B) and OVCA420 (C).
