# Supplementary material for: Combined treatment of disulfiram with PARP inhibitors suppresses ovarian cancer
Source: Front Oncol. 2023 Apr 18;13:1154073. doi: 10.3389/fonc.2023.1154073 (PMC10151711; doi:10.3389/fonc.2023.1154073)
Supplement: Supplementary file 2 [file Table_1.docx]

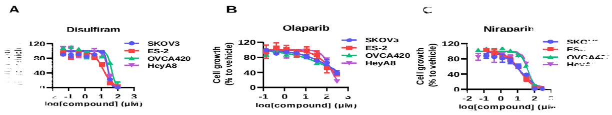

D

IC₅₀ (μM)			
Cell lines	Disulfiram	Olaparib	Niraparib
SKOV3	25.35± 1.24	189.40± 1.70	12.29± 1.24
ES-2	14.08± 1.96	152.50± 3.39	10.66± [illegible]
OVCA420	41.02± 0.49	129.57± 4.18	41.80± [illegible]
HEYA8	23.54± 1.34	145.35± 5.02	44.96± [illegible]
